# Vaccination as an Equaliser? Evaluating COVID-19 Vaccine Prioritisation and Compensation

**DOI:** 10.1093/medlaw/fwac020

**Published:** 2022-12-09

**Authors:** Christian Günther, Lauren Tonti, Irene Domenici

**Affiliations:** Max Planck Institute for Social Law and Social Policy, Munich, Germany; Max Planck Institute for Social Law and Social Policy, Munich, Germany; Max Planck Institute for Social Law and Social Policy, Munich, Germany

**Keywords:** COVID-19, Equity, No-fault compensation, Prioritisation, Vaccination, Vulnerability

## Abstract

This article assesses the equity of COVID-19 vaccination programmes in three jurisdictions that have historically taken different approaches to the institutionalisation of equity considerations. The Sars-Cov-2 pandemic has thrown into sharp relief persistent societal inequalities and has added novel dimensions to these problems. Certain groups have proved particularly vulnerable, both in terms of infection risk and severity as well as the accompanying social fallout. Against this background the implementation of ‘objective’ vaccination programmes may seem like a great leveller, addressing the disparate risks that are tied to social determinants of health and the pandemic behemoth. However, implementing vaccination programmes in an equitable manner is itself essential for the realisation of such a vision. This article undertakes a comparative analysis of the English, Italian, and American jurisdictions and critically assesses two aspects of their vaccination frameworks: (i) the prioritisation of groups for vaccination and (ii) the nature of public compensation schemes for those who have suffered vaccine-related injuries. It examines whether and to what extent these measures address the inequalities raised by COVID-19 and the role of the law in this pursuit.

## I. INTRODUCTION

The Marmot Review posited that ‘health inequalities that could be avoided by reasonable means are unfair’.^1^ COVID-19 provided responders the opportunity to address the disease’s well-documented disproportionate impact on societies’ most vulnerable. This applied especially to the deployment of vaccines which formed a significant aspect of the public health response to the pandemic. Though states can pursue many avenues to address COVID-induced health inequalities, national vaccination programmes serve as one of the most far-reaching and widely-adopted population health interventions available, and as such beg further examination.

This contribution argues that the implementation of these programmes and their congruence with the ideal of health equity was closely related to the legal frameworks in which they were embedded. Two specific dimensions of the COVID-19 vaccination process were selected for comparison, given the opportunities each present to infuse equity considerations in their design and implementation. First, vaccine prioritisation schemes could consider equity in allocation strategies adopted in times of vaccine scarcity. Second, vaccine injury compensation schemes redistribute resources to the subset of the population suffering from vaccine injury-induced disability. Incorporating COVID-19 into these schemes can address lack of confidence or access difficulties exacerbated by social disadvantage while simultaneously classifying the injured into a distinct category of vulnerability.

To test this hypothesis, this article examines three legal systems—England, Italy, and the USA—that embrace vaccination as a population health strategy, but have pursued different strategies to institutionalise equity considerations. It is argued that this variation shapes COVID-19 vaccination regulation and in turn demonstrates the advantages and disadvantages of the respective approaches. England has primarily adopted a procedural approach, whereby legal norms direct the attention of decision-makers to relevant equity considerations, but grants them discretion to determine the outcome of these deliberations. This is paired with a relatively limited system of substantive rights. Italy embraces a comprehensive substantive approach whereby the principle of equity goes hand in hand with that of social solidarity. Under Article 3 of the Constitution, all citizens are considered formally equal before the law and the state must give those who are disadvantaged the means to participate equally in social and political life. In contrast, the USA features an inherently ‘political’ approach, where the fifty federal states individually confront a socio-political ideal of equity without guidance from codified mandates.

These differentiated systems lend themselves to a comparative analysis, assessing how the different normative approaches shaped: (i) vaccine prioritisation programmes’ recognition of the greater need of some groups for the vaccine, as well as the need to overcome pre-existing patterns of disadvantage, and (ii) the realisation of vaccine injury compensation schemes’ objectives of meeting the needs of the injured and reinforcing public trust in vaccines.^2^

## II. THE ROLE OF VACCINATION AS AN EQUALISER IN THE PANDEMIC

### A. The Approach to Cross-Cutting Societal Inequities Exacerbated by the COVID-19 Pandemic

The pursuit of equity involves the diminishment of inequalities.^3^ Inequality in health implies differences in health, or significant influences thereon, that are ‘systematically associated with being socially disadvantaged’.^4^ Health inequalities encompass differences between socially advantaged groups and others in society,^5^ often resulting from differentials of power, money, resources, or prestige.^6^ Equity-oriented health measures should aim to reduce differences resulting from avoidable or unfair influences, and minimise differentials to the greatest extent possible.^7^

Comparison countries maintained different starting points in equity pursuits. England, generally provides universal access to healthcare through the National Health Service (NHS). Yet a decade of austerity measures and the implementation of anti-immigration policies meant that this country entered the pandemic with rising health inequalities.^8^ Similarly, while Italy offers (constitutionally guaranteed) equal access to healthcare, cultural, social, and administrative barriers have persisted. This heightened the risk of COVID-19 for migrants and asylum seekers.^9^ Overcrowding and low hygiene standards in prisons were also of significant concern.^10^ The USA contrasts with these (formally) universal approaches. Its minimalistic welfare state presents a history of restricted healthcare access and social injustice. The racial and ethnic disparities among COVID-19 cases, hospitalizations, and deaths can be traced to established intersectional dimensions of the social determinants of health.^11^

There are nevertheless common challenges across these states, with COVID-19 circumscribing the opportunities of historically disadvantaged populations to achieve health and well-being. Disparate COVID-related health outcomes corresponded with the following characteristics: (i) increased age;^12^ (ii) health conditions, including physical and learning disabilities;^13^ (iii) race/ethnicity;^14^ (iv) socio-economic disadvantage;^15^ and (v) membership among socially excluded groups, including prisoners,^16^ undocumented migrants/asylum seekers^17^ and the homeless.^18^ These give rise to an inequality in need that ought to be factored into equity-oriented health measures. As disparities based on these characteristics provide an added impetus for equitable responses, they form the principal focus of this article.

### B.*The Role of Vaccination as Equaliser*

Considering the above equity considerations and in response to these dangers, vaccination can act as an equalising agent. While no panacea, vaccination offers one of many avenues through which the state can address unequal distribution of societal resources and risk. Vaccination represents a scientifically-backed means for the state to minimise shared risks, and to protect and promote health through minimally invasive means.^19^ Vaccinations address health threats among the immunologically and socially vulnerable, by providing both individual and community-level protections. Finally, key vaccination services are also ordinarily made available through the welfare state at no or reduced cost in our three jurisdictions.

A comprehensive vaccination scheme involves a combination of interrelated elements, including measures that: ensure the availability of vaccines, enable universal access, promote vaccine uptake, and provide injury compensation. Equitable vaccination schemes can and should be designed to address potential inequalities within each of these dimensions. This article examines two key state-influenced elements of vaccination programmes to assess the factors that affected the realisation of this objective in our jurisdictions.

## III. COMPARING EQUITY CONSIDERATIONS IN ENGLAND, ITALY, AND THE UNITED STATES’ VACCINATION SCHEMES

### A. Vaccination Prioritisation

Initial COVID-19 vaccine scarcity presented nations with the difficult task of apportioning available doses. This often took the form of phased distribution, establishing prioritisation groups based upon defined characteristics of particular populations. This section examines how these schemes responded to equity considerations and how the characteristics of the respective legal systems shaped the process.

#### 1. England

In England, the Department for Health and Social Care (DHSC) delegates its responsibilities for delivering immunisation to NHS England, while maintaining strategic oversight.^20^ In this task it is aided by the *Joint Committee on Vaccination and Immunisation* (JCVI), an independent departmental expert committee with a basis in statute.^21^ The advice of this committee has become binding in some restricted instances, though the relevant conditions were not fulfilled for COVID-19.^22^ Consequently, the legal basis for prioritisation was the DHSC’s translation into policy of JCVI recommendations, in the course of which it performed its own equality impact assessment.^23^

Under this policy, the first priority group was those aged 80 years and over, older care home residents and those employed in the health and social care sector.^24^ Following this there was a stratification according to age and clinical vulnerability, with the *clinically extremely vulnerable* being equated with those aged 70–74 years and those *at risk* being prioritised after those aged 65–69 years.^25^ The overriding goal of this approach was to reduce harm and to ensure easy and quick deliverability, given that age and co-morbidities were taken as the strongest indicators of a heightened absolute risk.^26^ The vaccination of those occupied in the health and social care sectors was intended to provide a further benefit to these groups and an added society-wide benefit of maintaining well-functioning health and social care systems.

For those populations where evidence indicated that COVID-19’s impact was worsened by health inequalities, the increase in risk was said to be uncertain, subject to confounding variables and, in any case, smaller.^27^ The impetus to address these factors was thus reduced. The response to these wider health inequalities was also in conflict with the goal of designing a simple and speedily deliverable programme. Separating between sub-groups was argued to decrease the public understanding of the system and the trust in it.^28^ Singling out other groups for prioritisation was feared to reinforce negative stereotypes and to decrease their trust in vaccination, as it may be perceived as ‘experimental’.^29^ Further, dimensions such as membership of protected groups, ethnicity, or refugee status were poorly tracked.^30^ This complicated a response to health inequalities through vaccination and meant that these populations were not initially included in the national prioritisation programme. Instead, local decision-makers were given some operational flexibility to mitigate health inequalities in their areas.^31^

Egalitarian considerations were only realised in the design of the vaccination programme in so far as they were aligned with the above harm-reduction framework, i.e. concerning age and comorbidities. To the extent that the mitigation of inequalities was raised as a separate ‘principle’, it was only retrospectively addressed by two policy changes. The first change related to the prioritisation of those with learning disabilities. Only those with Down’s syndrome, those with ‘severe and profound’ learning disabilities and those in residential care had been prioritised at the national level. This created a distinction between those with mild/moderate and those with severe learning disabilities which was not readily captured in clinical records.^32^ This led some authorities to make use of their local flexibility to treat all persons on the learning disability register as ‘at risk’.^33^ A change in national approach followed in late February that placed all individuals with learning difficulties in the ‘at risk’ category.^34^

A second change concerned the homeless.^35^ Again, a national approach to prioritisation had not been envisioned. Nevertheless, homeless populations faced substantial risks of infection and poor health outcomes, given related co-morbidities. Moreover, many such co-morbidities were undiagnosed and could not be relied on as proxies for prioritisation.^36^ In response, local decision-makers again took the lead in prioritising these populations.^37^ Subsequently, following JCVI advice, the DHSC changed its policy in March. The homeless were included in the ‘at risk’ cohort.^38^ Despite such positive changes, other vulnerable groups were not prioritised in England, including: prisoners, asylum seekers, ethnic minorities, and the socio-economically disadvantaged.

The law’s role in the realisation of these equity-related objectives has been notable. Particularly that of the obligation on public actors to consider equality implications under the general Public Sector Equality Duty (PSED) contained in the *Equality Act* (EA) 2010.^39^ That this duty applies to the Secretary of State and the DHSC is placed beyond doubt by their specification in the EA.^40^ The JCVI, in contrast, is unlisted and subject to the duty only when performing a public function.^41^ In 2018 it had denied this regarding the provision of vaccination recommendations.^42^ However, this was challenged under the PSED,^43^ and the JCVI conceded the point in its COVID-19 vaccination advice, recognising its ‘legal duty to prevent discrimination based on protected characteristics’.^44^ Given the public interest in the prioritisation scheme for the COVID-19 vaccine, and the closeness with which the Committee worked with the DHSC in delivering the government’s policy objectives, this is undoubtedly the correct conclusion.^45^

Consequently, the PSED required the Secretary of State and the JCVI: to ‘have due regard to the need to […] advance equality of opportunity between persons who share a relevant protected characteristic and persons who do not share it’.^46^ Such regard must be given before the decision.^47^ The relevant, currently covered protected characteristics are age, sex, disability, and race.^48^ It is clear that an authority may have to consider affording preferential treatment to one of these protected groups, if its needs are different.^49^ Thus, insofar as the old and disabled, who had a heightened need, were given preferential access to the vaccine, this was supported by the legal framework. Insofar as the disparate needs of other protected groups were unmet, it is to be noted that the PSED leaves considerable discretion to decision-makers:

The concept of ‘due regard’ requires the court to ensure that there has been a proper and conscientious focus on the statutory criteria, but if that is done, the court cannot interfere with the decision simply because it would have given greater weight to the equality implications of the decision than did the decision maker.^50^

Although this duty may be further heightened where a large vulnerable population is affected,^51^ the JCVI’s detailed and responsive elaboration of equity considerations arguably falls within this discretion. By comparison, the Secretary of State did not appear to have paid them any regard. Only after the initiation of legal proceedings—concerning the decision not to prioritise all those with learning disabilities—was an impact assessment produced. Ultimately this satisfied the High Court that the relevant considerations had been present, and led to a refusal of permission for judicial review.^52^ This indicates that, for this highly sensitive political decision, the PSED did direct decision-makers to important health equity considerations, although it does not provide a tool for stricter legal oversight.

#### 2. Italy

Italy has a prominent legal and political culture concerning the universal provision of vaccination, extending to the constitutional permissibility of population-wide vaccine mandates.^53^ Due to COVID-19 vaccine scarcity, the initial approach was not to use this power,^54^ but to pursue a staggered vaccination campaign that would ultimately guarantee free population-wide access. This campaign was organised according to the prioritisation criteria presented to Parliament by the Minister of Health in December 2020,^55^ and then adopted via Ministerial Decree in January 2021. The strategic plan was developed by an interdisciplinary working group set up at the Ministry of Health, together with the Special Commissioner for the COVID-19 emergency, The Italian National Institute of Health, the Italian National Agency for Regional Healthcare Services and the Italian Medicines Agency.^56^ The working group consulted with the National Bioethics Committee. The plan was explicitly based on core constitutional values and especially on the right to health of the individual and the community. When submitting it to Parliament, the Minister announced that ‘no inequalities will be allowed in the vaccination campaign’.^57^

As an initial phase, the plan provided for the prioritisation of healthcare professionals as well as staff and residents of residential care facilities and all elderly over 80.^58^ A second stage of the campaign, implemented in February 2021, envisaged a prioritisation based on health conditions. Clinical vulnerability to COVID-19 was primarily identified with advanced age, but also with the co-morbidities contained in the vaccination plan—including Down’s syndrome and enumerated severe physical or mental disabilities.^59^ Simultaneously, the plan provided for a ‘parallel track’ of vaccinations exclusively using vector vaccines,^60^ which were then only recommended for people under 55. This permitted vaccination based less on clinical conditions than certain social considerations. In particular, it was envisaged that educational staff, the armed forces and the police, prison staff and inmates, and staff working in civil and religious communities or carrying out ‘other essential activities’ could be vaccinated.^61^ The plan’s implementation was entrusted to the health administrations of the individual regions.

Parallel prioritisation criteria sought to address some of the aforementioned inequalities. Above all, the inclusion of prison staff and inmates improved the vulnerable conditions of the prison population during the pandemic.^62^ In addition, the prioritisation of educational staff prompted the resumption of school activities, mitigating the problems posed by the so-called ‘digital divide’.^63^ However, this implementation brought into focus other inequalities. Vaccinating the young and less clinically vulnerable before some of the elderly generated feelings of inequality and injustice.^64^ Moreover, the vague reference to ‘other essential activities’ posed interpretation problems that exacerbated certain inequalities. Some regions prioritised socio-economically advantaged groups, including judges, lawyers, and journalists, thus creating forms of discrimination that the National Committee for Bioethics has described as ‘ethically relevant’.^65^

As a result of these challenges, a further update of the vaccination plan in March resolved the ambiguity around ‘other essential activities’. The update removed the distinction between vector and RNA vaccines and considered that all people with severe disabilities, as defined by Law no. 104/1992, were vulnerable.^66^

As a state action, the vaccine plan must be constitutionally compatible. The need to protect the functioning of the national healthcare system—and thus the right to health as a public interest (Article 32)—justified the prioritisation of medical personnel. In relation to equity, Article 3(2) mandates that state measures must guarantee substantive equality in circumstances of unequal starting conditions. The prioritisation of more vulnerable individuals—those of advanced age or those with comorbidities—was no doubt an adequate instrument to achieve this equity goal.^67^ Similarly, prioritising individuals belonging to areas particularly affected by the pandemic, such as education and prisons, contributed to achieving substantive equality under Article 3. The category of persons performing ‘other essential activities’ did not contribute to the goal of restoring equality because of its vagueness, which left room for divergent interpretations. It was a missed opportunity to effectively protect other factually disadvantaged groups. Additional prioritisation for other vulnerable groups such as ethnic minorities, asylum seekers or the homeless could have been constitutionally permissible.

#### 3. United States

COVID-19 vaccination prioritisation in the USA is marked by a significant lack of codified law with which to guide it. Despite this, vaccination efforts attempted to at least consider health inequalities, even if the ultimate result is insufficient. In the USA, a federal republic in which many public health powers belong to states unless federally pre-empted, each state maintains responsibility for developing its own COVID-19 vaccination distribution plan.^68^ Through unique mechanisms, such as state agency task forces or advisory panels, these jurisdictions determined COVID-19 vaccine allocation strategies, often based on non-binding guidelines issued by the federal government or national entities. Two key guideline-issuing entities are the National Academies of Sciences, Engineering, and Medicine (NASEM) and the Advisory Committee on Immunization Practices (ACIP).

Both NASEM and ACIP considered equity in their suggested initial vaccine prioritisation strategies, and emphasised avoiding disparity exacerbation or creation, though each suggested different methods of doing so.^69^ In its *Framework for Equitable Allocation of COVID-19 Vaccine*, NASEM explicitly acknowledges that vaccine allocation schemes should address COVID-related inequities ‘rooted in structural inequalities, racism, and residential segregation’ as a moral imperative.^70^ ACIP, upon whom CDC relies to make immunisation recommendations, similarly set forth goals of disparity reduction in light of COVID-19 inequity data.^71^

While plans suggested ways to incorporate equity considerations into scarce vaccine distribution, they remained non-legally binding suggestions. Though many states mirrored ACIP recommendations, fidelity to NASEM and ACIP guidelines was not absolute. After guidance issuance, vaccine prioritisation fell victim to the classic federal public health response challenge in the USA—heterogeneity.^72^ Deviation from the guidelines among states was especially pronounced when it came to equity considerations. Almost every state included frontline healthcare and long-term care workers as first priority populations, largely in accord with ACIP recommendations.^73^ ACIP next prioritised essential workers, ranking this population partly based on the reported high incidence among members and ‘the disproportionate impact of COVID-19 on workers who belong to racial and ethnic minority groups’.^74^ ACIP names justice and health inequity mitigation among the ethical principles that support this prioritisation, acknowledging essential workers’ risk of exposure and that vaccination ‘minimis[es] societal disruption and prevent[s] morbidity and mortality’.^75^ However, not all states prioritised essential workers next, some opting to prioritise teachers, those with elevated health risks, or the elderly after healthcare workers.^76^ While long-term care facility staff, who may come into contact with people with disabilities, were initially first prioritised, those living with disabilities themselves were variably prioritised.^77^ States that prioritised disabled populations did so according to ranging definitions of the term, at times including those living with intellectual and developmental disabilities, physical disabilities, mental or behavioural health conditions, or some combination of these categories in the definition.^78^ Incarcerated and homeless populations were similarly nonuniformly prioritised among states.^79^

By using disadvantage indices, some states accounted for potential socially derived vulnerabilities in their COVID-19 vaccination plans. Disadvantage indices are statistical measures that seek to capture the relative average disadvantage of a population living in a particular geographic area. Relevant metrics include income, education level, and housing quality.^80^ These indices, such as the Social Vulnerability Index (SVI), Area Deprivation Index (ADI), and COVID-19 Community Vulnerability Index (CCVI), can all help address inequality in COVID contexts, capturing populations to which immunity is most required, in light of the ability to social distance or monitoring areas experiencing disparate impacts.^81^ A survey of state vaccination plans shows that thirty-seven jurisdictions committed to using some type of disadvantage index.^82^ Twenty-nine jurisdictions refer to SVI in their prioritisation plans, while the thirteen remaining utilise other indices.^83^ NASEM recognised vulnerability indices as valuable means to accomplish equity objectives in a conscious, evidence-based way.

An index tool that can account for racial/ethnic inequity better accords with the jurisdiction’s existing constitutional precedents. States could face significant constitutional challenges if they used individuals’ race or ethnicity as primary factors in prioritisation decisions.^84^ While no precedent for allocating scarce health resources based on race exists, race-based classification has been reviewed in the context of other public goods under strict scrutiny.^85^ This requires that governmental measures be narrowly tailored to achieve a compelling interest. Considering other Supreme Court precedents, using only race/ethnicity as the sole determining allocation factor will likely fail a strict scrutiny test.^86^ However, incorporating race/ethnicity as one of numerous data points in a community-level index is likely to face a lower level of scrutiny and would consequently prove less challenging.^87^

Still, without a positive, unifying legal framework each state acted independently in their approach to vaccine allocation. Even states that chose to utilise vulnerability indices did not always employ the same index, nor did they employ indices to the same ends. Some states employed disadvantage indices to apportion vaccines or vaccination appointments. Other states committed to reserving percentages of vaccine allocations to areas with high disadvantage scores, specifically referring to SVI or CCVI.^88^ Still others weighted allocations using SVI metrics to increase allocations to disadvantaged populations.^89^

Despite stated equity commitments and planning considerations, inequalities persisted, with data revealing lower vaccination rates in non-White communities.^90^ While epidemiological factors played a predominant role in US states’ prioritisations, some social aspects also mattered. Prominent guidance mentioned equity considerations, though a lack of binding legal obligation allowed for divergence and discrepancy.

### B. Vaccination Injury Compensation Schemes

England, the USA, and Italy feature public no-fault payment mechanisms for those injured by vaccines. These too can be analysed in terms of the outlined equity objectives. Most directly these schemes constitute a state’s response to disparate needs that have arisen from a public health measure benefitting society as a whole. Addressing vaccine injury-induced disability responds to the fact that the inevitable ‘burden of injury itself is unequally distributed’ among the population.^91^ Another objective that policymakers pursue through these mechanisms is to increase vaccine confidence. Under this approach, the position of vaccine-hesitant populations should grant additional significance to the task of designing ‘trustworthy’ schemes. Reassuring these groups through a remedy in case of injury could encourage equitable *utilisation*.

#### 1. England

Following the Report of the Royal Commission on Civil Liability and Compensation for Personal Injury (the Pearson Report), the *Vaccine Damage Act* (VDPA) *1979* was enacted to create the first public scheme of payments for vaccine damage in England: The Vaccine Damage Payment Scheme (VDPS).^92^ Although this has evolved it remains in force today. For present purposes, a significant change has been the addition of COVID-19 to the list of specified diseases to which the act applies.^93^ Additionally, for COVID-19 vaccines there was an abrogation of the condition that the person receiving the vaccine must (generally) be under 18.^94^ The exhaustive listing of such diseases, the absence of wider legal criteria for their identification,^95^ and the discretionary relaxation of certain requirements, exemplify the essentially political basis for the scheme.^96^

Access to the scheme is relatively uncomplicated, with the submission of a form to the relevant agency.^97^ Beyond this, the VDPA imposes two principal limitations on claims. First, it only provides remedies to those who suffer death or severe disablement from vaccination.^98^ ‘Severe’ encompasses ‘disablement to the extent of 60 per cent’—assessed according to wider social security principles.^99^ Second, the scheme requires proof of causation on a balance of probabilities.^100^ This corresponds to ordinary civil law claims—which are not precluded^101^ – but, unlike such claims, it does not require proof of fault. Upon a successful claim, the Secretary of State can award a statutory sum of up to £120,000 in a single payment.^102^ No special provisions are made for the funding of this.

The VDPS was proposed as a political solution to the exigencies of a particular factual situation. It was a countermeasure to the waning trust in childhood vaccination programmes.^103^ Hereby the particular vulnerability of children and the need to assist families became prominent considerations in the inception of the scheme.^104^ A need to signal confidence in vaccination and to increase vaccine uptake was central to the VDPA’s creation.^105^ In discussing the impact on vaccination uptake, the Pearson Commission considered that ‘the Government must be confident about vaccination before it would make such provision’.^106^ An understanding of the Act as a political, fact-specific response is further supported by its characterisation as an ‘immediate’ or ‘interim’ measure by the Labour Government, which intended to follow up with a more comprehensive scheme.^107^ However, this prospect was forestalled by the Labour Party’s election loss in 1979.

Recognising the role of external circumstances is not to deny that socio-political ideals underpinned the VDPA. Through its implementation, Parliament recognised a distinct degree of responsibility for the vaccine injury based on: the public benefit derived from vaccinations;^108^ the moral responsibility that stemmed from state-endorsed vaccination programmes,^109^ and/or the desire to spread the cost of the resulting hardship across the community.^110^ The pursuit of these goals helps to explain the outlined nature of the scheme, especially why its remedy is not compensatory in the sense of corresponding to the loss suffered.^111^ The statutory sum is a limited recognition of the state’s moral responsibility for the injury, intended only to alleviate distress and to provide a measure of assistance to the injured and their carers. Providing true compensation was explicitly left to the common law courts.^112^ Consequently, the availability of independent civil law claims is central to the proper functioning of the scheme, though notably restricted in the context of COVID-19 vaccines.^113^

In assessing the law’s influence on this scheme one may ask whether the *European Convention on Human Rights* (ECHR) provides a legal framework for assessing the scope of the VDPS’s coverage. Did it mandate that COVID-19 be added to the list of specified diseases? Could it be used to challenge persistent distinctions drawn by the VDPS, for instance between those who have similar needs but are deemed to be more/less than 60% disabled?^114^ Two cases suggest that the ECHR is ill-suited to play this role. In *Godfrey v United Kingdom*^115^ the VDPA was claimed to be in breach of Articles 8 and 14 by compensating only those suffering injuries after (but not before) 1948—i.e. by drawing an unjustified distinction between disabled individuals with comparable needs. This claim was declared inadmissible by reference to the Convention’s limited scope relating to social security measures. Recently the Court of Appeal echoed this approach in *R (on the application of CN) v Secretary of State for Health and Social Care*.^116^ While this did not concern the VDPA itself, it involved a challenge to a similar compensation scheme that excluded the claimant’s illness from a list of admissible conditions.^117^ Given that the Secretary of State was ‘concerned with both judgments of social and economic policy and with disability’ the court accepted that they would be subject to a ‘wide margin of appreciation’ in justifying limitations on the scheme.^118^ Although the issue of disability discrimination was thus recognised, the ECHR cannot meaningfully determine the scheme’s coverage under the current deferential approach.

In contrast, the PSED under the EA proactively informed the extension of the VDPS to COVID-19. This sought to achieve equity and enhance public trust by putting COVID-19, with its greater impact on the oldest age groups, on equal footing with other vaccines.^119^ Further, the immediate application to adults must also be seen in the context of a 2018 concession that the PSED had been breached by not taking this step for influenza vaccination.^120^ This legal mechanism thus strengthened the English scheme’s emphasis on encouraging vaccine uptake and it framed the decision to meet the needs of one especially vulnerable group.

Overall, the consideration of the PSED was, nonetheless, perfunctory. There was no mention of the benefit the VDPS could provide to other at-risk groups, including certain ethnicities, and those with pre-existing disabilities. Wider changes to the VDPS in light of COVID-19 were also curtly dismissed by reference to political and time constraints.^121^ In this respect, this process contrasts unfavourably with the assessments of vaccination prioritisation. While a more comprehensive consideration may not be legally mandated (see section III.A.1.), closer regard to the legal framework could have directed decision-makers to a more consistent, equitable response.

#### 2. Italy

The Italian no-fault compensation scheme is provided for by Law no. 210/1992. It was only explicitly extended to encompass COVID-19 vaccinations in late January 2022. Article 20(1) of Decree-law 27th January 2022, no. 4, provides that compensation shall be paid to ‘those who have suffered injury or disability, which resulted in a permanent impairment of psycho-physical integrity, due to vaccination against SARS-CoV-2 recommended by the Italian Health Authority’. Article 20(1-bis) allocated €50 million to this scheme for the year 2022 and €100 million per year starting from 2023.

Regarding the access conditions and remedy, it must be noted that the scheme implements an ‘equitable system of compensation’ which does not aim to provide recompense for the damage suffered.^122^ Such an award can still be pursued through civil law.^123^ In comparison with such a claim, a victim only has to prove causality between the vaccine and the damage—and not fault.^124^ The causality requirement is generally judicially interpreted as a criterion of ‘reasonable scientific probability’, whereby judges can base their decision on a preponderance of the evidence and the consideration that a causal link is ‘more likely than not’.^125^ However, some cases and commentators have indicated that the causation requirement is to be assessed less stringently in no-fault compensation cases than in civil liability cases. The mitigation of the burden of proof and a less stringent causation assessment allegedly arise from the social solidarity function of this instrument.^126^ Albeit controversial, this approach is part of a wider judicial trend.^127^

Upon a successful claim, Law no. 210/1992 grants a lifelong measure of support,^128^ the amount of which is established by reference to eight categories of compensation that correspond to the severity of the injury.^129^ For 2021, the monthly sum ranges from €803,68 to €899,98.^130^ Moreover, Law no. 229/2005 grants an additional monthly living allowance to be split between the injured subject and related carers. Depending on the category of damage, this is four to six times the amount granted by Law no. 210/1992.

The nature of this remedy accommodates the demands of the Constitutional Court, which repeatedly stated that the sum cannot be purely trivial^131^ and shall consist of an ‘equitable’ compensation. Its precise amount is nonetheless subject to statutory determination^132^ and its constitutional legitimacy could only be doubted in the extreme case of the compensation being so negligible as to *de facto* nullify the right itself.^133^

The extension of the compensation scheme laid down in Law no. 210/1992 to include COVID-19 vaccines was not a pure act of political will. While the government may have eventually decided to take action, it was also obliged to do so by substantive principles of the constitutional order, as elaborated by the Constitutional Court. Had the government further delayed in enacting such regulation, the same outcome would have eventually been brought about by the Constitutional Court itself. A survey of constitutional case-law in this matter clarifies this conclusion.

Against the background of constitutionally permissible compulsory vaccination policies,^134^ judgement no. 307/1990 of the Constitutional Court first obliged the legislature to provide no-fault compensation for vaccine injuries. The Court arrived at this conclusion primarily by weighing two aspects of Article 32: the protection of health as a fundamental right of the individual and as a major interest of the community.^135^ To guarantee both elements, the legislature was to provide an equitable system of compensation for vaccine-injured individuals. Law no. 210/1992 responded to this and provided the contemporary basis for the vaccination compensation scheme.^136^ A right to public compensation was granted to anyone who had suffered permanent impairment of their psycho-physical integrity as a result of vaccinations made compulsory by law or by order of a health authority.^137^ The wording of the Act limited the right to compensation to irreversible damages caused by compulsory vaccinations.

Yet the Constitutional Court continued to specify the nature of the relevant obligation. The first step was its holding that the legislature’s duty must be viewed in light of Article 2.^138^ Hereby, individuals are required to fulfil fundamental duties of political, economic and social solidarity. Individuals receiving a vaccination fulfil a duty of solidarity by acting in the collective interest and for the protection of others, while incurring a risk to themselves. As a result, the community is, in return, bound to the individual by this duty.^139^ The right to compensation is thus understood to be based on reciprocal solidarity, and on the consideration that withholding compensation from an individual who has sacrificed themselves for the public good would be contrary to a principle of justice.^140^ It would be unfair and unconstitutional to impose a solidarity obligation from which only one side would benefit.^141^

Utilising this theory, the Court has expanded the statutory scheme—holding that fair compensation must also be provided in the case of certain recommended vaccinations.^142^ As an immediate effect of these judgments, the compensatory scheme was extended to damages from vaccinations that are (only) strongly recommended by the health authorities. The purported distinction between compulsory vaccinations and those ‘merely’ recommended could not justify a differentiation in the support of those who may be harmed by them.^143^ The principle of solidarity operates with the same force in both scenarios. It would be irrational to compensate those who were compelled by law to be vaccinated while denying it to those who voluntarily received it out of considerations of social solidarity and public benefit—which were suggested by the government campaign.^144^ The payment scheme was consequently extended without legislative mediation: Law no. 210/1992 remained in force, but specific non-compulsory vaccines could be brought into its scope.

The most recent judgement, no. 118/2020, specified this case law.^145^ It emphasised that the compensation regime can only be extended to newly recommended vaccinations upon explicit consideration by the Constitutional Court, which must check for the fulfilment of certain criteria.^146^ In particular, it must assess whether the campaign was suited to instil in citizens a sense of duty towards safeguarding public health in the interest of the community and confidence in the recommended vaccine.^147^ This clarification was initially applicable to COVID-19. In fact, until the enactment of Decree-law 27th January 2022, no. 4, only people belonging to a category for which vaccination was compulsory (i.e. healthcare workers and, later, individuals over the age of 50) could successfully apply for compensation. For the remainder of the population, a prior submission to the Constitutional Court would have been necessary. However, the intense awareness campaigns launched by the government, as well as the quasi-coercive instruments put in place to fight the pandemic,^148^ left the outcome of such a case in little doubt.^149^ It is against this background that, in January 2022, the government finally rectified its unconstitutional omission by explicitly extending the scheme to COVID-19 vaccinations.

#### 3. United States

The National Vaccine Injury Compensation Program (VICP) and the Countermeasures Injury Compensation Program (CICP) are the two key no-fault vaccination compensation schemes in the USA. Aiming to balance efficient and fair compensation avenues for those affected by vaccines with vaccine manufacturers’ protection from liability, VICP compensates those who experience injury by a covered vaccine, one that the CDC recommends for routine use among children or pregnant women and is subject to a federal excise tax.^150^ The Vaccine Injury Table lists eligible vaccinations and enumerates the potential injuries associated with a vaccine’s administration, as well as the time period in which such conditions manifest.^151^ If a petitioner’s symptoms match table conditions, reviewers can presume causal association. For unlisted injuries, the petitioner must prove the vaccine caused the injury.^152^

CICP, the second no-fault compensation scheme, is authorised by the Public Readiness and Emergency Preparedness Act (PREP Act). Activated during public health emergencies, CICP compensates for injuries related to countermeasures administered to counteract health security threats, a category under which vaccinations fall.^153^ Barring wilful misconduct, the PREP Act grants tort claim immunity under federal and state law for countermeasure manufacturers, distributors, prescribers, administrators, dispensers, or programme planners.^154^ CICP petitioners may need to present significant scientific evidence to make their cases for causation, as temporal association alone may be insufficient to prove injury. Only listed injuries create ‘rebuttable presumptions’ that a countermeasure caused the injury.^155^

The origin of federal legislation that gives rise to VICP and CICP can be traced back to focusing events that elevated the need for vaccine-related recourse. In the 1980s, focus heightened on parents’ litigation against pharmaceutical companies for their children’s alleged adverse health events shortly following diphtheria, pertussis, and tetanus (DPT) vaccination.^156^ Simultaneously, Congress heard testimony from a variety of stakeholders on immunisation safety.^157^ Manufacturers’ hesitation, ignited by uncertainties related to pending legislation, evolving jurisprudence, potential windfall tort awards, and difficulties in acquiring liability insurance resulted in manufacturers leaving the market, and ultimately shortages for certain vaccines.^158^ Created through the National Childhood Vaccine Injury Act of 1986, VICP attempts to balance recourse with liability protection against windfall judgements and litigation costs.^159^ CICP’s origins relate to focusing events involving national security. The PREP Act, was passed in the wake of the September 11 terrorist attacks and the fear of bioterrorism.^160^ CICP, a PREP Act component, ensures the supply of medical interventions, like vaccines, in exchange for manufacturer liability protection.

Rather than include COVID-19 vaccines in VICP, pandemic-related vaccination remains extraneous to the traditional system. During public health emergency declarations, like the COVID PREP Act declaration in March 2020, claims for compensation due to adverse vaccination events are routed to CICP.^161^ Importantly, CICP petitioners cannot later recover under VICP, even after the emergency.^162^ While other PREP Act aspects have been periodically amended during the pandemic, the legislation has not been amended to expand compensation. To date, COVID-related injuries are not listed in statutory Covered Countermeasures Injury Tables.^163^

Arguably, access considerations were seeded in political will for the ultimate passage of compensation schemes through congressional legislation. Given the nation’s experience with vaccine supply disruption, market availability relied on balancing compensation for harm and liability protection. VICP and CICP solutions provide tort protection incentives for a consistently available vaccine supply, thus enabling access. With litigation mounting and no state duty to ensure vaccination supply, Congress realised the role of vaccines in ensuring health and welfare and acted. VICP motivations allude to an understanding of fairness that gives deference to pharmaceutical manufacturers. Solidarity against a common threat could also be inferred. However, such schemes seem to operate under the assumption that risk and access are distributed evenly among the population. Given the significant body of health equity research, especially in light of COVID-19, that assumption is inadequate.

Despite similarity in nomenclature and no-fault conceptions, VICP and CICP differ in their petition conditions and compensation award.^164^ In terms of qualifying injury, CICP covers only serious physical injury and death whereas VICP covers not only deaths, but also injuries with effects lasting for six months or more following vaccine administration, as well as hospitalisation or surgery.^165^ CICP is also criticised for being a less generous compensation programme. CICP reimburses for ‘reasonable and necessary’ medical expenses, as well as some lost employment income.^166^ Lost employment income is limited to 66 ⅔ percent of the injured’s unreimbursed gross income at the time of injury, up to $50,000 for lost employment annually until the age of 65 years.^167^ Projected future earnings are not considered in the calculation.^168^ While some medical expense payments are allowed without limit,^169^ there is no compensation for pain and suffering, rehabilitation, special education, or other therapies, among other restrictions.^170^ An injury under the VICP scheme, however, awards petitioners actual and projected non-reimbursable expenses for medical care, rehabilitation, residential care, behavioural healthcare, support services and other related expenses without limit.^171^ Additionally, VICP can award up to $250,000 for actual and projected pain and suffering and emotional distress, as well as lost earnings and ‘reasonable’ lawyers’ fees and legal costs.^172^ CICP does not reimburse for legal fees, whereas relief may be available in qualified VICP cases.^173^

CICP’s processes, in which applicants have limited opportunities to participate in determinative agency proceedings, are non-transparent.^174^ Moreover, CICP proceedings are clandestine, as petitions are privately assessed by department officials, without time limits or interaction between officials and petitioners.^175^ Without published CICP decisions, the public is not privy to the evaluation of adverse events linked to a vaccine.^176^ VICP, a judicial process, offers judicial avenues for appeal, whereas CICP, an administrative process, offers a one-time administrative reconsideration.^177^ Together, these differences indicate that, generally, awards for COVID-related vaccine injury will be less generous, compared to non-emergent schemes, after a lengthy, expensive, and non-transparent administrative process pursuing compensation.^178^

## IV. NORMATIVE EVALUATIONS OF VACCINATION AS AN EQUALISER

### A. Vaccine Prioritisation

Each of the three approaches to vaccine prioritisation sought to address health inequities in the distribution of vaccine access. One commonality is that age, correlated co-morbidities and healthcare occupation were consistently prioritised in all of them. Further, the three countries all made some provision for the disabled. The precise nature of this category and the urgency of vaccination were not consistent, however. While England and Italy both placed certain disabled individuals relatively high on their national priority lists, the former created a problematic distinction between those who were severely and moderately disabled. The distinct needs—both clinical and social—of those deemed moderately disabled were not initially met. Only after a failed legal challenge and after local actors began to prioritise this group was this changed. Italy’s fine-grained approach to eligible disabilities may be similarly critiqued and, it too, had to amend its policy. Hereby it implemented a more comprehensive approach in early March, which could draw on an established statutory definition. The USA did not provide for a consistent national approach at all. The term disability was variably defined in the context of COVID vaccination, leading to inconsistent treatment across state lines. Consequently, the response to this vulnerable group’s need was variable, limited and liable to be adjusted.

Such variance is even more pronounced when one looks at the handling of the other socially disadvantaged populations. Across and within the three states no consistent prioritisation approach can be identified. In the USA, national advice encouraged federal and local decision-makers to consider race, ethnicity, and socio-economic status in the allocation of vaccines. In the absence of legal obligations, this was only ever considered in some localities. Some states also went beyond national advice, placing especially vulnerable groups—including the homeless and prisoners—into high priority categories. This highlights the overall heterogeneity of response and of upholding equity as a value. Beneficial local approaches could also be found in England, albeit to a much more limited extent, in the initiative that select local authorities took in the prioritisation of the homeless and which ultimately led to a change in national strategy. By comparison, the local dimension of the Italian scheme was distinctly negative. While the criterion of ‘essential activities’ had the potential to benefit some vulnerable groups indirectly, this was variably and counterproductively applied and ultimately abolished. The national scheme compared favourably, however, by granting prisoners priority-status early on.

The legal frameworks structuring these categories exerted a varied and limited influence across all nations, despite the importance of the issues at stake. The USA provides a stark example of how the substantive requirements of constitutional law constrained their consideration. The US Supreme Court’s precedent of applying strict scrutiny to policies based solely on explicit racial or ethnic distinctions discouraged states from directly incorporating these groups’ disparate needs into prioritisation decisions. Though the law did not prohibit the incorporation of more indirect measurements or a direct focus on other particularly vulnerable groups, the patchwork of prioritisation attests that those positive decisions were much more attributable to political will than to legal norms in the USA. The emergence of such a patchwork highlights the fickleness of relying on political norms over procedural or substantive options, which can lead to further social fission along lines of disadvantage.

Italy’s substantive constitutional framework had a positive, albeit limited, influence on the implementation of the vaccination plan. Admittedly, the government strategy for prioritisation responded to constitutionally recognised needs. This was reflected in: the prioritisation of those with a higher clinical need for the vaccine, the consideration of wider social determinants of health, and the abolition of the ‘essential activities’ category, whose implementation was constitutionally suspect. However, the constitutional principle of equity could not provide concrete standards for the extension of the vaccination plan to other disadvantaged groups. Here the Constitution left room for considerable political discretion, especially in light of the initial vaccine scarcity. Beyond implicitly informing the vaccination scheme, Italy’s system of substantive rights therefore also ultimately left the pursuit of an equitable response to political actors.

Under the English framework, it was not substantive rights, but legal requirements for a process-driven incorporation of equity considerations that influenced vaccine prioritisation, with decision-makers conducting relevant assessments. Moreover, the legal challenges initiated under this duty contributed to an important body (the JCVI) recognising its relevant obligations and to an amendment of the national prioritisation scheme in favour of individuals with learning disabilities. English law did not allow for successful challenges of specific decisions, but it furthered the realisation of equity objectives in the political sphere.

Overall, there were significant limitations in the ways that each of the prioritisation programmes tackled health inequalities. This was demonstrated not only by the variations and amendments within states, but especially by the different responses to comparable problems across them. Unfortunately, legal frameworks did relatively little to counteract these deficiencies: they did not demand consistent or comprehensive approaches to health inequities. Here there is definite room for improvement.

### B. Vaccine Injury Compensation

An equitable vaccination campaign not only involves a fair distribution of vaccines but also requires the encouragement of utilisation and a response to the unequal burdens (vaccine injury-induced disability) that are created from their deployment. The examined compensation schemes purportedly pursue these interrelated goals, and yet it will be argued that the legal frameworks of the Anglo-Saxon countries are ill-suited to this task. In contrast, the Italian scheme, informed by a substantive right of solidarity, requires an equitable redistribution of resources and provides the requisite certainty, comprehensiveness, and consistency to instil public confidence.

For COVID-19 the pursuit of these objectives initially meant that coverage had to be extended to this novel virus. This occurred in all jurisdictions, but in distinct ways. In the USA, coronavirus undoubtedly fell under a bespoke legislative scheme designed for emergencies, providing coverage for potential injuries. Similarly, while Italy only has a single scheme, its constitutional jurisprudence set out clear, forward-looking criteria that provided the requisite reassurance. Even before the extension to COVID-19, this guaranteed that injuries from state-encouraged vaccines would be compensable. By contrast, the English scheme was the most uncertain. Lacking a pre-existing means of extending it to COVID-19, prompt executive action was necessary to pre-empt doubts regarding coverage.

The programmes’ wider entitlement conditions speak directly towards the group of vaccine-injured individuals that will receive support for their situation. In all jurisdictions, the removal of a fault requirement ensures that greater coverage is achieved than by the ordinary judicial process. This is complemented by the prospect that the ‘easy availability of compensation increases willingness to undergo vaccination’.^179^ For this purpose a programme must not only be in place, but it must be seen to cover deserving individuals and it must also be accessible to disadvantaged groups.

The American and English programmes fall short in the realisation of these aims. The procedural hurdles under the US scheme pose distinct problems for accessibility, impacting petitioners who lack access to legal or financial resources. In addition, both jurisdictions impose restrictive entitlement conditions. Most significantly they only compensate for death or serious injury. Needs that may be significantly heightened but fall below this threshold go unmet. As with prioritisation, an arbitrary distinction is created between different kinds of disability and, in both jurisdictions, this is conceptualised as a political decision with little to no legal direction. In particular, the unsuccessful claims under the ECHR illustrate the limited nature of the UK’s substantive rights and, in contrast with vaccine prioritisation, its procedural approach has effected little. It did precipitate an extension of the scheme to older age groups and it did lead decision makers to posit a connection between the scheme and confidence in the COVID-19 vaccines. Yet there was no thorough examination of other vulnerable populations, vaccine hesitancy and the potentially positive effects of further relaxations. This was said to be precluded by practical political considerations.

In comparison, the Italian scheme is much broader, flowing from a constitutional obligation of social solidarity. As this obligation applies equally to all vaccine-damaged individuals, the legislation lists even relatively minor forms of damage as compensable harms. Insofar as the schemes are intended to achieve a redistribution based on unequal need, the law has played a remarkably positive role, mandating a truer approximation to this ideal. This framework also means that an individual is assured that if they act in solidarity by participating in the state’s vaccination policy they will be supported should they suffer injury as a result.

Causation requirements pose a special problem for schemes that seek to assist the vaccine injured and encourage trust in vaccination. If the causation standard is set too high, it may exclude many deserving individuals, potentially impacting the public’s perception of the scheme—especially since causation is notoriously difficult to establish for vaccine injuries. Arguably this is evidenced in England, and to a lesser extent the USA, where restrictive causation requirements noticeably impact the success of claims.^180^ If the standard is set too low, however, the success of spurious claims can make vaccine risks appear greater than they are, so that compensation schemes could negatively impact public trust.^181^ Italy exposes itself to this danger, given that some courts have eased the relevant requirements. Moreover, the offsetting benefits for public confidence are limited by the fact that this represents a case-law driven approach whose contestability has consequences for transparency and predictability.^182^ Nevertheless, this relaxation of causation is influenced by the objectives underlying the Italian compensation scheme, including the realisation of its social support function. To this extent (without evaluating compensatory justice) it represents a preferable model. It creates a space for the law to frame these considerations and prevents an unduly onerous causation requirement from effectively undermining the equity-related objectives of such schemes.

Permissible access conditions only go so far towards generating public trust or meeting this group’s needs. One must also regard the remedy. Here again the Italian model stands out. Multiple generous awards are provided as continuous payments to support the vaccine-injured. Both England and the USA offer more nominal awards, with England being easily the least generous. These sums do not comprehensively or continuously meet individual needs. Awarding immunity from civil law claims to relevant private parties adds another problematic dimension. Whereas the operation of these schemes is usually premised on the ‘backstop’ of such claims, this has been denied under present circumstances.

Vaccination compensation schemes purport to marry the goals of meeting the injured’s needs and encouraging vaccine uptake. Of the three nations, Italy’s approach, shaped by its legal framework, is best suited to achieve this aim. The Italian Constitution determined the compensation scheme’s creation, its extension to COVID-19, and the provision of adequate remedies. The USA and England compare poorly. Although the U.S. scheme also provided certainty regarding the coverage of COVID-19, both have restrictive access conditions and offer only limited remedies, England in particular. Ultimately, the fact that the Anglo-Saxon schemes are political creations permitted a neglect of equity considerations. It remains an ongoing task to make decision-makers aware of them and to precipitate positive legislative changes.

## 5. CONCLUSION

In a pandemic that has highlighted so much socially derived inequity, stakeholders had the opportunity to ameliorate inequities in their COVID-19 vaccination campaigns. These were reasonable means of addressing profound societal challenges and it was seen that England, Italy, and the USA grappled with this task. This article highlighted the role of the law in this pursuit. Even amongst schemes with overlapping objectives, there was not one ideal framework. Where wider legal norms had a limited influence, this admittedly contributed to variable responses and dissonance in the realisation of relevant aims, as is exhibited especially by the U.S. and English schemes. Yet for the vital task of vaccine prioritisation, the latter’s light-touch procedural approach was a valuable mechanism that directed political decision-makers towards the implementation of equity considerations. The Italian Constitution, with its strong substantive rights, offered little guidance for this sensitive dimension, while playing a central role in creating a coherent and comprehensive vaccine injury compensation scheme. While basic legal institutions may not be readily transplantable, their respective advantages and drawbacks ought to be recognised. To this end, this article has sought to illustrate what is possible in the pursuit of equitable vaccination campaigns.

*Conflict of interest statement*. None declared.

## FUNDING

The authors did not receive funding for this work.

## Footnotes

1 Michael Marmot and others, ‘Fair Society, Healthy Lives - The Marmot Review’ (Institute of Health Equity 2010) 3.

2 This article does not evaluate the empirical evidence that compensation schemes increase confidence in vaccines. It discusses confidence in reference to the normative ideas and motivations behind instituting compensation schemes, which as been documented. See section III.B.1. below and Martin Keane and others, ‘Vaccine Injury Redress Programmes: a Systematic Integrative Review’ (2019) 24 BMJ Evid-Based Med A7–A8.

3 Paula Braveman, ‘Health Disparities and Health Equity: Concepts and Measurement’ (2006) 27 Ann Rev Public Health 167–94.

4 ibid.

5 ibid.

6 ibid; Marmot and others (n 1) 16.

7 Margaret Whitehead, ‘The Concepts and Principles of Equity and Health’ (*WHO Regional Office for Europe* 1990) 7 <https://citeseerx.ist.psu.edu/viewdoc/download?doi=10.1.1.196.7167&rep=rep1&type=pdf> accessed 7 June 2022.

8 Michael Marmot and others, ‘Health Equity in England: The Marmot Review 10 years on’ (Institute of Health Equity, 2020); Caitlin Boswell, ‘“We also want to be safe”: Undocumented Migrants Facing COVID in a Hostile Environment’ (The Joint Council for the Welfare of Immigrants 2022).

9 Massimo Fabiani and others, ‘Epidemiological Characteristics of COVID-19 Cases in Non-Italian Nationals Notified to the Italian Surveillance System’ (2021) 31(1) Eur J Public Health 37, 40; Ifeoma Nneka Emelurumonye and Alessandro Miglietta, ‘New Arrival Migrants and Asylum Seekers in Reception Centres in Italy During COVID-19’ (30 July 2020) <https://migrationhealth.org/wp-content/uploads/2021/05/lancet-migration-situational-brief-italy-02-en.pdf> accessed 7 June 2022. For the impact of the pandemic on socio-economically vulnerable groups, see Laura Palazzani, ‘La giustizia e i criteri etici per la distribuzione dei vaccini nell’ambito della pandemia Covid-19: linee della discussione bioetica’ (2021) 2 BioLaw J – Rivista di BioDiritto 137, 147–48.

10 See *Torreggiani and Others v Italy*, app nos 43517/09, 46882/09, 55400/09, 57875/09, 61535/09, 35315/10 and 37818/10 (ECtHR, 8 January 2013); *Sulejmanovic v Italy*, app no 22635/03 (ECtHR, 16 July 2009); Emilio Dolcini, ‘Carcere e COVID-19: Una Cronistoria dal decreto “cura Italia” al “decreto ristori”’ (2021) 1 Rivista Italiana di Diritto e Procedura Penale 3, 6; Sophie Charlotte Monachini, ‘Carcere e Covid-19: pena arcaica, emergenza nuova: Il sistema penitenziario italiano in tempo di pandemia’ (2021) 5 Diritto Penale e Uomo <https://dirittopenaleuomo.org/wp-content/uploads/2021/04/Monachini_DPU.pdf> accessed 7 June 2022; Piergiorgio Fedeli and others, ‘L’epidemia da covid-19 nelle carceri’ (2020) Riv Ita Med Leg 837, 839–40.

11 Jarvis T Chen and others, ‘Intersectional Inequities in COVID-19 Mortality by Race/Ethnicity and Education in the United States, January 1, 2020–January 31, 2021’ (23 February 2021) Harvard Center for Population and Development Studies Working Paper 21 (3) <https://www.hsph.harvard.edu/population-development-dev/wp-content/uploads/sites/2623/2021/02/21_Chen_covidMortality_Race_Education_HCPDS_WorkingPaper_Vol-21_No-3_Final_footer.pdf> accessed 7 June 2022; National Academies of Sciences, Engineering and Medicine (NASEM), *Framework for Equitable Allocation of Covid-19 Vaccine* (The National Academies Press 2020).

12 Public Health England, ‘Disparities in the Risk and Outcomes of COVID-19’, PHE Publication (Public Health England 2020).

13 Public Health England, ‘Deaths of People Identified as Having Learning Disabilities with COVID-19 in England in the Spring of 2020’, PHE Publication (Public Health England 2020).

14 Public Health England, ‘Beyond the Data: Understanding the Impact of COVID-19 on BAME groups’ PHE Publication (Public Health England 2020); Fabiani and others (n 9) 40; Roberto Baronio and others, ‘Impact of Ethnicity on COVID-19 Related Hospitalizations in Children During the First Pandemic Wave in Northern Italy’ (2021) 9 Front Pediatr 1; Chen and others (n 11); NASEM (n 11).

15 Michael Marmot and others, ‘Build Back Fairer: The COVID-19 Marmot Review. The Pandemic, Socioeconomic and Health Inequalities in England’ (Institute of Health Equity 2020) 13; NASEM (n 11).

16 Associazione Antigone, ‘Oltre il Virus: XVII Rapporto di Antigone sulle condizioni di detenzione’ (May 2021) <https://www.rapportoantigone.it/diciassettesimo-rapporto-sulle-condizioni-di-detenzione/> accessed 7 June 2022. See also Dolcini (n 10); Chantal Edge and others, ‘COVID-19 and the Prison Population’, Working paper for the COVID-19 impact inquiry (Health Foundation 2021); Eddie Burkhalter and others, ‘Incarcerated and Infected: How the Virus Tore through the U.S. Prison System’ (*New York Times*, 10 April 2021) <https://www.nytimes.com/interactive/2021/04/10/us/covid-prison-outbreak.html?auth=login-email&login=email&smid=url-share> accessed 7 June 2022.

17 Emelurumonye and Miglietta (n 9); Felicity Knights and others, ‘Impact of COVID-19 on Migrants’ Access to Primary Care and Implications for Vaccine Roll-Out: a National Qualitative Study’ (2021) 71 Br J Gen Pract J Royal Coll Gen Pract e583; Kathleen R Page and others, ‘Undocumented U.S. Immigrants and Covid-19’ (2020) 382 N Engl J Med e62.

18 Dan Lewer and others, ‘COVID-19 among People Experiencing Homelessness in England: a Modelling Study’ (2020) 8 Lancet Resp Med P1181.

19 Lawrence O Gostin, ‘Mandatory Vaccination: Understanding the Common Good in the Midst of the Global Polio Eradication Campaign’ (2018) 7 (4) Israeli J Health Policy Res.

20 The National Health Service Act 2006, s 7A; the NHS Public Health Functions (‘Section 7A’) Agreement 2020/21; Elizabeth Rough, *UK Vaccination Policy* (Briefing Paper, Number CBP 9076, 2021) 4.

21 The National Health Service (Standing Advisory Committees) Order 1981, SI 1981/597; Joint Committee on Vaccination and Immunisation (JCVI), *Code of Practice*, June 2013.

22 Health Protection (Vaccination) Regulations 2009, SI 2009/38, reg 2; Elizabeth Cleaver, ‘Briefing 976: The Complexity of Public Law Challenges during the Pandemic – Seeking Priority Access to the Covid-19 Vaccine for People with Learning Disabilities’ (2021) Discrim Law Assoc Brief 14, 15.

23 ibid 17.

24 Department for Health and Social Care (DHSC), *UK COVID-19 Vaccines Delivery Plan* (2021), para 4.2.

25 ibid section 4, table 2.

26 Joint Committee on Vaccination and Immunisation (JCVI), *Annex A: COVID-19 Vaccine and Health Inequalities: Considerations for Prioritisation and Implementation* (6 January 2021) <https://www.gov.uk/government/publications/priority-groups-for-coronavirus-covid-19-vaccination-advice-from-the-jcvi-30-december-2020/annex-a-covid-19-vaccine-and-health-inequalities-considerations-for-prioritisation-and-implementation> accessed 7 June 2022.

27 ibid.

28 ibid.

29 ibid.

30 ibid.

31 DHSC (n 24) para 4.5.

32 Keri-Michèle Lodge, Christian Brown and Sheila Hollins, ‘People with an Intellectual Disability should be Prioritised for Vaccination’ (*BMJ Opinion*, 14 January 2021) <https://blogs.bmj.com/bmj/2021/01/14/people-with-an-intellectual-disability-should-be-prioritised-for-vaccination/> accessed 7 June 2022.

33 Gareth Lacobucci, ‘Covid-19: GPs OPT TO Prioritise all Patients with Learning Disabilities for Vaccination’ (2021) BMJ 372: n510.

34 Department for Health and Social Care, ‘Letter from the Health and Social Care Secretary on COVID-19 vaccination in people with learning disabilities: 23 February 2021’ (24 February 2021) <https://www.gov.uk/government/publications/letter-from-the-health-and-social-care-secretary-on-covid-19-vaccination-in-people-with-learning-disabilities/letter-from-the-health-and-social-care-secretary-on-covid-19-vaccination-in-people-with-learning-disabilities-23-february-2021> accessed 7 June 2022.

35 JCVI (n 21).

36 Department for Health and Social Care, ‘Letter from the JCVI to the Health and Social Care Secretary on Further Considerations on Phase 1 Advice: 1 March 2021’ (11 March 2021) <https://www.gov.uk/government/publications/letter-from-the-health-and-social-care-secretary-on-covid-19-vaccination-phase-1-advice/letter-from-the-jcvi-to-the-health-and-social-care-secretary-on-further-considerations-on-phase-1-advice-1-march-2021> accessed 7 June 2022.

37 Awil Mohamoud, ‘Councils and GPs Prioritise Covid Vaccinations for Homeless People and Rough Sleepers’ (15 January 2021) <https://managementinpractice.com/covid-19/councils-and-gps-prioritise-covid-vaccinations-for-homeless-people-and-rough-sleepers/> accessed 7 June 2022.

38 Department for Health and Social Care, ‘Letter from the Health and Social Care Secretary to the JCVI: 11 March 2021’ (11 March 2021) <https://www.gov.uk/government/publications/letter-from-the-health-and-social-care-secretary-on-covid-19-vaccination-phase-1-advice/letter-from-the-health-and-social-care-secretary-to-the-jcvi-11-march-2021> accessed 7 June 2022.

39 Equality Act 2010, part 11.

40 ibid s 150(1) and schedule 19 part 1.

41 ibid s 149(2).

42 Landmark Chambers, ‘HPV Vaccine Set to be Extended to Boys, after Pressure on Equality Grounds’ (24 July 2018) <https://www.landmarkchambers.co.uk/resource-post/hpv-vaccine-set-to-be-extended-to-boys-after-pressure-on-equality-grounds/> accessed 7 June 2022.

43 ibid.

44 JCVI (n 21).

45 Relevant factors are outlined here: The Equality and Human Rights Commission, Technical guidance on the public sector equality duty: England (2021) para A.11.

46 Equality Act 2010 sn 149(1)(b).

47
*R* *(on the application of BAPIO Action Ltd) v Secretary of State for the Home Department* [2007] EWCA Civ 1139 [3] (Sedley LJ).

48 Equality Act 2010 section 4.

49
*R* *(on the application of Kaur) v Ealing LBC* [2008] EWHC 2062 (Admin).

50
*R* *(on the application of Hurley) v Secretary of State for Business, Innovation and Skills* [2012] EWHC 201 (Admin) [78] (Lord Justice Elias).

51
*R* *(on the application of Hajrula) v London Councils* [2011] EWHC 448 (Admin) [62] (Calvert Smith J).

52 Cleaver (n 22) 17.

53 This dates back to pre-constitutional legislation (anti-diphtheria vaccination was made compulsory by Law no 891/1939).

54 In a second stage, compulsory vaccination was introduced for healthcare professionals in April 2021 with Decree-law no 44/2021 and then extended in early 2022 to all citizens over 50 (Decree-law no 1/2022).

55 Senato della Repubblica. Assemblea, *Resoconto Stenografico*. XVIII Legislatura, 279ª seduta, 2 December 2020 <https://www.senato.it/service/PDF/PDFServer/BGT/1185376.pdf> accessed 7 June 2022.

56 ibid.

57 ibid.

58 ‘Vaccinazione anti-SARS-CoV-2/COVID-19: Piano strategico. Elementi di preparazione e di implementazione della strategia vaccinale’ (12 December 2020) G.U. no 72, 24 March 2021 (Serie Generale) 24, 29.

59 ‘Vaccinazione anti-SARS-CoV-2/COVID-19: Raccomandazioni ad interim sui gruppi target della vaccinazione anti-SARS-CoV-2/COVID-19’ (8 February 2021) attached to Ministry of Health, circolare 8.02.2021. The plan contained two tables listing several comorbidities according to which patients would fall into either the first priority group (extremely vulnerable patients, eg with respiratory diseases requiring oxygen therapy, kidney diseases requiring dialysis, cardiovascular diseases with heart failure, certain types of diabetes, Down's syndrome, etc) or the fourth (other respiratory diseases, HIV, high blood pressure, etc).

60 Viral vector vaccines use modified viruses as avenues for vaccines. This technology was used by the Oxford/AstraZeneca vaccine and the Janssen Pharmaceuticals vaccine, see: Andre Ian Francis and others, ‘Review of COVID-19 Vaccine Subtypes, Efficacy and Geographical Distributions’ (2022) 98 Postgrad Med J 389–94.

61 ‘Vaccinazione anti-SARS-CoV-2/COVID-19: Raccomandazioni ad interim’ (n 59).

62 Dolcini (n 10) 29–30; Palazzani (n 9) 146; Comitato Nazionale per la Bioetica (CNB), ‘Urgenza vaccinale: Aspetti bioetici’ (12 March 2021) <https://bioetica.governo.it/media/4133/m25_2021_urgenza-vaccinale-aspetti-bioetici.pdf> accessed 7 June 2022.

63 Palazzani (n 9) 146; Comitato Nazionale per la Bioetica (CNB), ‘COVID-19 e bambini: dalla nascita all’età scolare’ (23 October 2020) <https://bioetica.governo.it/media/4114/p139_covid-19-e-bambini-dalla-nascita-all-eta-scolare_it.pdf> accessed 7 June 2022.

64 Francesca Coin, ‘Le disuguaglianze del piano vaccinale in Italia’ (*Internazionale*, 23 March 2021) <https://www.internazionale.it/opinione/francesca-coin/2021/03/23/disuguaglianze-piano-vaccinale-italia> accessed 7 June 2022; CNB (n 63) 3.

65 Coin (n 64); CNB (n 63) 4.

66 ‘Vaccinazione anti-SARS-CoV-2/COVID-19: Raccomandazioni ad interim sui gruppi target della vaccinazione anti-SARS-CoV-2/COVID-19’ (10 March 2021), adopted with Ministerial Decree of 12 March 2021.

67 Giuseppe Battarino ‘Prime riflessioni su criteri costituzionalmente fondati di distribuzione dei vaccini anti-Sars-Cov-2’ (*Questione Giustizia*, 24 November 2021) <https://www.questionegiustizia.it/articolo/prime-riflessioni-su-criteri-costituzionalmente-fondati-di-distribuzione-dei-vaccini-anti-sars-cov-2> accessed 7 June 2022.

68 Harald Schmidt and others, ‘Equitable Allocation of Covid-19 Vaccines in the United States’ (2021) 27 (7) Nat Med 1298; Harald Schmidt and others, ‘Is It Lawful and Ethical to Prioritize Racial Minorities for Covid-19 Vaccines?’ (2020) 324 (20) JAMA 2023–24.

69 Schmidt and others, ‘Equitable Allocation’ (n 68).

70 NASEM (n 11) 95.

71 Kristen Underhill and Olatunde CA Johnson, ‘Vaccination Equity by Design’ (2021) 131 (53) Yale Law J Forum 53, 65–67.

72 ibid 67–68.

73 J Raifman and others, ‘COVID-19 US State Policy Database’ (*Boston University*, 2020) <https://statepolicies.com/data/graphs/vaccine-rollout/?id=4> accessed 7 June 2022; Johns Hopkins University, ‘State Vaccination Plans’ <https://coronavirus.jhu.edu/vaccines/vaccine-state-plans> accessed 7 June 2022; Kathleen Dooling and others, ‘The Advisory Committee on Immunization Practices’ Interim Recommendation for Allocating Initial Supplies of COVID-19 Vaccine — United States, 2020’ (2020) 69 (49) MMWR 1857.

74 Kathleen Dooling and others, ‘The Advisory Committee on Immunization Practices’ Updated Interim Recommendation for Allocation of COVID-19 Vaccine — United States, December 2020’ (2021) 69 (5152) MMWR, 1657.

75 ibid.

76 ibid; Jennifer Kates, Jennifer Tolbert, and Josh Michaud, ‘The COVID-19 “Vaccination Line”: An Update on State Prioritization Plans’ (*Kaiser Family Foundation*, 11 January 2021) <https://www.kff.org/coronavirus-covid-19/issue-brief/the-covid-19-vaccination-line-an-update-on-state-prioritization-plans/> accessed 7 June 2022.

77 Johns Hopkins Disability Health Research Center, ‘Vaccine Prioritization Dashboard’ (21 April 2021). <https://disabilityhealth.jhu.edu/vaccine-2/> accessed 7 June 2022; MaryBeth Musumeci and Priya Chidambaram, COVID-19 Vaccine Access for People with Disabilities’ (*Kaiser Family Foundation*, 1 March 2021) <https://www.kff.org/medicaid/issue-brief/covid-19-vaccine-access-for-people-with-disabilities/> accessed 7 June 2022.

78 Johns Hopkins Disability Health Research Center (n 77).

79 Kaiser Family Foundation, ‘State COVID-19 Vaccine Priority Populations’ (19 April 2021) <https://www.kff.org/other/state-indicator/state-covid-19-vaccine-priority-populations/?currentTimeframe=0&sortModel=%7B%22colId%22:%22Location%22,%22sort%22:%22asc%22%7D> accessed 7 June 2022; Rachel Strodel, and others, ‘Covid-19 Vaccine Prioritization of Incarcerated People Relative to Other Vulnerable Groups: An Analysis of State Plans’ (2021) 16 (6) PLoS One e0253208.

80 Schmidt and others, ‘Equitable Allocation’ (n 68).

81 ibid.

82 ibid.

83 ibid.

84 Govind Persad, ‘Allocating Medicine Fairly in an Unfair Pandemic’, (2021) 3 Univ Illinois Law Rev 1085, 1096.

85 Schmidt and others, ‘Is It Lawful’ (n 68).

86 ibid; Persad (n 84).

87 Schmidt, and others, ‘Is It Lawful’ (n 68); Persad (n 84) 1122–23.

88 Schmidt and others, ‘Equitable Allocation’ (n 68).

89 ibid.

90 Underhill and Johnson (n 71) 54, 62–63; Uché Blackstock and Oni Blackstock, ‘Opinion: White Americans are being Vaccinated at Higher Rates than Black Americans. Such Inequity Cannot Stand’ *Washington Post* (1 February 2021) <https://www.washingtonpost.com/opinions/2021/02/01/racial-inequality-covid-vaccine/> accessed 7 June 2022.

91 Michelle M Mello, ‘Rationalizing Vaccine Injury Compensation’ (2008) 22 Bioethics 32, 40.

92 Colin Pearson, *Report of the Royal Commission on Civil Liability and Compensation for Personal Injury* (1978) vol 1.

93 The Vaccine Damage Payments (Specified Disease) Order 2020, SI 2020/1411, art 2.

94 ibid art 3.

95 Vaccine Damage Payments Act 1979, s 1(2).

96 Gareth Millward, ‘A Disability Act? The Vaccine Damage Payments Act 1979 and the British Government’s Response to the Pertussis Vaccine Scare’ (2017) 30 Soc Hist Med 429.

97 See: ‘Vaccine Damage Payment’ (GOV.UK) <https://www.gov.uk/vaccine-damage-payment/how-to-claim> accessed 7 June 2022 and The NHS Business Services Authority (Awdurdod Gwasanaethau Busnes y GIG) (Vaccine Damage Payments Scheme) Directions 2021.

98 Vaccine Damage Payments Act, s 1(1)(a).

99 Vaccine Damage Payments Act, s 1(4); Social Security (General Benefit) Regulations 1982, SI 1982/1408, sch 2. The courts have applied the exclusively physical criteria to non-physical injuries: *Secretary of State for Work & Pensions v FG* [2017] EWCA Civ 61 [45-46].

100 Vaccine Damage Payments Act, s 3(5).

101 ibid s 6(4).

102 ibid ss 1-1(A) and The Vaccine Damage Payments Act 1979 Statutory Sum Order 2007, SI 2007/1931.

103 Millward (n 96) 440–41; Pearson (n 92) 293.

104 Pearson (n 92) 294; HC Deb 9 May 1978, vol 949, cols 974–976.

105 HC Deb 5 February 1979, vol 962, col 62.

106 Pearson (n 92) 298.

107 Millward (n 96) 443; Stephanie Pywell, ‘A Critical Review of the Recent and Impending Changes to the Law of Statutory Compensation for Vaccine Damage’ (2000) JPI Law 246, 250; HC Deb 5 February 1979, vol 962, col 38.

108 HC Deb 5 February 1979, vol 962, col 43.

109 HC Deb 6 June 2000, vol 351, col 11WH.

110 HC Deb 9 May 1978, vol 949, col 977.

111 ibid col 976.

112 ibid.

113 The Human Medicines (Coronavirus and Influenza) (Amendment) Regulations 2020, SI 2020/1125, reg 29.

114 Pywell (n 107) 253–54.

115 App no 8542/79 (ECommHR, 4 February 1982).

116 [2022] EWCA Civ 86.

117 A scheme ‘the very existence and scope of [which] was pre-eminently a discretionary decision for the Secretary of State taking the cost, the moral obligation and the fault-based backdrop into account’: ibid [36].

118 ibid [45].

119 Department for Health and Social Care (DHSC), *Expansion of the Vaccine Damage Payment Scheme (VDPS) for COVID-19* (Impact Assessment, IA No 9564, 2020).

120 This was in response to legal proceedings: ‘Government Agrees to Rethink Vaccination Compensation Age-Bar After Admitting Equality Rule Breach’ (*Hodge Jones and Allen*, 19 March, 2018) <https://www.hja.net/news-and-insights/press-releases/personal-injury/government-agrees-to-rethink-vaccination-compensation-age-bar-after-admitting-equality-rule-breach/> accessed 7 June 2022.

121 DHSC (n 119).

122 Paola Frati and others, ‘No-Fault Compensation and Anti-COVID-19 Compulsory Vaccination: The Italian Context in a Broad View’ (2022) 10 Vaccines 635.

123 Rosanna Breda, ‘Danno da vaccinazione tra indennizzo e risarcimento del danno’ (2018) Riv It Med Leg 296.

124 Cassazione, sez. lav., judgement no 7354/2021, see Fulvia Passananti, ‘Le vaccinazioni obbligatorie o raccomandate ed il diritto all’indennizzo’ (2021) 21 (2) AmbienteDiritto 1, 12; Andrea Cioni, ‘La corsa al vaccino contro il COVID-19. Qualche considerazione fra requisiti per l’autorizzazione e regole di responsabilità’ (2020) Resp civ e prev 2017, 2024; Breda (n 123) 293; Andrea Volpato, ‘Nesso causale e danno da vaccinazione: è tempo per una rilettura della legge n. 210/1992?’ (2021) Resp civ e prev 1167.

125
*Inter alia* Cassazione, sez lav, decision no 24959/2017 and Cass, sez lav, judgement no 2474/2021. See Stefano D'Errico, ‘Autismo e vaccinazioni, un pericoloso passo indietro della giurisprudenza di merito e un monito per il consulente tecnico dell'Ufficio. La criteriologia: rigore metodologico o orpello di insufficienza?’ (2010) Riv It Med Leg 526.

126 See Trib Treviso, sez lav, no 176/2010, commented in Andrea Parziale, ‘Danno da vaccinazione e incertezza del nesso causale: il ruolo della prova per presunzioni’ (2017) Riv Ita Med Leg 1067. The necessity to apply a less stringent standard in the case of non-fault compensation, due to its social support function, is invoked also by Volpato (n 124) 1177. See also Eleonora Ranjeri and others, ‘Remedies for Damage Caused by Vaccines: A Comparative Study of Four European Legal Systems’ (2018) Eur Rev Private Law 61–63.

127 Cassazione civ judgement no 2474/2021. See Volpato (n 124) 1174, Parziale (n 126) 1075–76.

128 Cassazione civ judgement no 13923/2000. See Breda (n 123) 292.

129 Art 2.1, Law no 210/1992. See Breda (n 123) 293.

130 Eight different categories of impairment to physical integrity are defined in ‘Tabella A’ attached to the DPR no 834 of 30 December 1981. Among the least severe injuries, the table lists mouth injuries leading to swallowing or speech disorders, persistent cardiac functional disorders, chronic otitis, mild but persistent neurosis and others. The most serious impairments include injuries causing incapacity to work, deafness, injuries to the central nervous system with serious consequences, loss of several limbs.

131 Italian Constitutional Court (ICC) judgement no 118/1996.

132 ICC judgements nos 307/1990, 118/1996 and 27/1998.

133 ICC judgement no 27/1998.

134 ICC judgements nos 307/1990 and 118/1996.

135 ICC judgement no 307/1990, para 2; Cristina Videtta, ‘Corte Costituzionale e indennizzo per lesioni alla salute conseguenti a trattamenti vaccinali. Nuove Prospettive’ (2013) Resp civ e prev 1030.

136 Camera dei Deputati. Commissione XXII Affari Sociali, *Resoconto Stenografico* X Legislatura, 77ª seduta, 29 January 1992 <http://legislature.camera.it/_dati/leg10/lavori/stencomm/12/Leg/Serie010/1992/0129/stenografico.pdf> accessed 7 June 2022. See also Breda (n 123) 289.

137 Art 1, Law no 210/1992.

138 ICC judgement no 118/1996, para 5.

139 Nicoletta Vettori, ‘Le decisioni in materia di salute tra precauzione e solidarietà. Il caso delle vaccinazioni’ (2018) Diritto Pubblico 181, 189.

140 ICC judgements no 118/1996 para 5, no 268/2017 para 6, no 118/2020 para 3.4.

141 Videtta (n 135) 1034.

142 ICC judgements nos 27/1998; 423/2000; 107/2012; 268/2017;118/2020.

143
*Inter alia*, judgements no 27/1998, para 3 and no 107/2012, para 3.

144 ICC judgement no 27/1998, para 3. See Roberto Caranta, ‘Danni da vaccinazione e responsabilità dello Stato (Nota a Corte Costituzionale, 26 febbraio 1998, n.27)’ (1998) Resp civ e prev 1352.

145 Donatella Morana, ‘Obblighi e raccomandazioni in tema di vaccinazioni, tra discrezionalità legislativa ed estensione del diritto all’equo indennizzo (in nota a Corte cost., sent. n. 118/2020)’ (2021) Osservatorio Costituzionale 233, 234.

146 ICC judgement no 118/2020 para 2. See Morana (n 145) 249; Andrea Parziale, ‘Vaccinazione raccomandata antiepatite A: il ruolo della giurisprudenza costituzionale nell'estensione della tutela indennitaria’ (2020) Riv Ita Med Leg 2135, 2143.

147 ICC judgement no 118/2020 paras 2 and 3.4. See Morana (n 145) 240.

148 Certification of COVID-19 vaccination is required to access several activities and services, see eg Decree-law no 105/2021.

149 Passananti (n 124) 23–26; Cioni (n 124) 2024.

150 42 US Code § 300aa–10; Katharine Van Tassel, Carmel Shachar, and Sharona Hoffman, ‘Covid-19 Vaccine Injuries — Preventing Inequities in Compensation’ (2021) 384 (10) N Engl J Med e34; Lainie Rutkow and others, ‘Balancing Consumer and Industry Interests in Public Health: The National Vaccine Injury Compensation Program and Its Influence During the Last Two Decades’ (2007) 111 (3) Dickinson Law Rev 681; 42 CFR § 100.3; 26 US Code §§ 4131, 4132.

151 42 CFR § 100.3.

152 42 US Code §§ 300aa–11, 300aa-14.

153 42 USC §§ 247d-6d(b), 247d-6d(i)(1).

154 42 USC § 247d-6d(i)(2).

155 42 CFR § 110.100; 42 CFR 110.20(b) and (c).

156 Rutkow and others (n 150).

157 ibid.

158 ibid 682.

159 National Vaccine Injury Compensation Program: Rescission of Revisions to the Vaccine Injury Table, 86 Fed Reg 21209-21213 (April 22, 2021); Rutkow and others (n 150).

160 Peter H Meyers, ‘The Trump Administration’s Flawed Decision on Coronavirus Vaccine Injury Compensation: Recommendations for Changes’ (2020) 7 (1) J Law Biosci; Lincoln Mayer, ‘Immunity for Immunizations: Tort Liability, Biodefense, and Bioshield II’ (2007) 59 (6) Stanford Law Rev 1753.

161 Declaration Under the Public Readiness and Emergency Preparedness Act for Medical Countermeasures Against COVID-19, 85 Fed Reg 15198-15203 (17 March 2020).

162 Van Tassel, Shachar, Hoffman (n 150).

163 42 CFR § 110.100.

164 Van Tassel, Shachar, Hoffman (n 150).

165 42 CFR § 110.3(z); 42 CFR § 247d-6e (3)(e); 42 USC § 300aa–15.

166 2 CFR §§ 110.30, 110.31.

167 42 CFR § 110.81(c) (1-4).

168 42 CFR § 110.81(a)(1).

169 42 CFR § 110.80.

170 42 CFR § 110.31(c); Meyers (n 160).

171 42 USC § 300aa–15.

172 ibid.

173 Health Resources & Services Administration (HRSA), ‘Comparison of Countermeasures Injury Compensation Program (CICP) to the National Vaccine Injury Compensation Program (VICP)’ (April 2021) <https://www.hrsa.gov/cicp/cicp-vicp> accessed 7 June 2022; Meyers (n 160).

174 ibid.

175 Meyers (n 160).

176 ibid.

177 ibid.

178 Van Tassel, Shachar, Hoffman (n 150).

179 Mello (n 91) 35. Though empirically contested, this remains an objective of policy makers and the schemes’ design should lend itself to this pursuit.

180
*Loveday v Renton* [1990] 1 Med LR 117 highlights many difficulties in establishing causation. In England the majority of claims fail on this basis: Duncan Fairgrieve, ‘Compensation for Vaccine Damage in the United Kingdom’ (2018) 19 Le Journal de Droit de la Santé et de l’Assurance Maladie 87; Richard Goldberg, ‘Vaccine Damage Schemes in the US and UK Reappraised: Making them Fit for Purpose in the Light of Covid-19’ (2022) Legal Stud 1.

181 Betsy Grey, ‘The Plague of Causation in the National Childhood Vaccine Injury Act’ (2011) 48 Harvard J Legislation 343.

182 Cf Goldberg (n 180) 18.

